# A cross-sectional study on social functioning and its associated factors among patients with severe mental illness in Minhang District, Shanghai

**DOI:** 10.3389/fpsyt.2026.1864002

**Published:** 2026-07-03

**Authors:** Jing Zhao, Yu Zhou, Ling Wang, Yihua Jiang, Weibo Zhang

**Affiliations:** 1 Department of Psychiatry, Shanghai Minhang Mental Health Center, Shanghai, China; 2 Shanghai Institute of Infectious Disease and Biosecurity, Fudan University, Shanghai, China; 3 Department of Maternal, Child and Adolescent Health, School of Public Health, Fudan University, Shanghai, China; 4 Department of Psychiatry, Shanghai Mental Health Center, Shanghai Jiao Tong University School of Medicine/National Medical Center for Mental Disorders, Shanghai, China

**Keywords:** anxiety symptoms, community management, depressive symptoms, severe mental illness, social functioning, social support

## Abstract

**Objective:**

To examine the current status of social functioning and its associated factors among patients with severe mental illness (SMI) in Minhang District, Shanghai, and to provide evidence for community-based rehabilitation interventions.

**Methods:**

A district-wide population-based cross-sectional survey was conducted in 2022 among all registered patients with SMI in Minhang District, Shanghai. Social functioning, perceived social support, anxiety symptoms, and depressive symptoms were assessed using the Social Disability Screening Schedule (SDSS), the Perceived Social Support Scale (PSSS), the Generalized Anxiety Disorder 7-item scale (GAD-7), and the Patient Health Questionnaire 9-item scale (PHQ-9), respectively. Univariate comparisons were performed using t-tests and ANOVA, with Bonferroni correction for *post-hoc* comparisons. Multiple linear regression, adjusted for age and gender, was used to examine factors independently associated with social functioning; multicollinearity was assessed using variance inflation factors (VIF).

**Results:**

A total of 3510 eligible patients were included. The prevalence of social dysfunction was 36.6%, with social interaction as the most severely affected domain. Depressive symptoms were more prevalent than anxiety symptoms, and 11.3% of patients had comorbid anxiety and depressive symptoms. After adjusting for age and gender, more severe depressive symptoms were independently and positively associated with social dysfunction (*P <* 0.001), whereas higher perceived social support was independently and negatively associated with social dysfunction (*P <* 0.001).

**Clinical significance:**

More severe depressive symptoms were associated with greater social dysfunction among patients with SMI, while higher social support was associated with better social functioning. Routine screening and timely intervention for depressive symptoms may be considered in community mental health management.

**Methodological innovation:**

This district-wide census using standardized clinical assessments provides a replicable model for regional mental health surveys in other large cities in China.

**Public health value:**

Integrating depressive symptom screening into routine follow-up may help improve the efficiency of regional mental health services and promote social functioning recovery among patients with SMI.

## 1 Introduction

Severe mental illness (SMI) (1) comprise a group of psychiatric disorders characterized by substantial functional disability. They include schizophrenia, bipolar disorder, mental disorders due to epilepsy, intellectual disability accompanied by mental disorders, paranoid psychosis, and schizoaffective disorder, among others. These disorders substantially impair cognitive, emotional, and behavioral functioning, and often result in marked deficits in social functioning. Social dysfunction is manifested as difficulties in personal daily living, fulfillment of family roles, participation in social interactions, and performance of occupational functions, and it is a key factor affecting patients’ reintegration into society.

With the continuous improvement of China’s mental health service system, rehabilitation of social functioning has received increasing attention. As a leading region in mental health practice, Shanghai has established a relatively mature prevention, treatment, and rehabilitation system. Minhang District, as a typical population-importing urban area, has a large base of SMI patients, making patient service management and community rehabilitation particularly challenging and creating an urgent demand for rehabilitation services. However, there is still a lack of systematic survey data on the social functioning of registered SMI patients in this district, and studies conducting district-wide, multidimensional evaluations remain absent. Therefore, our study adopted a cross-sectional design to assess the current status of social functioning among SMI patients registered in the Shanghai Mental Health Information System for Minhang District, and to analyze the effects of demographic characteristics, treatment status, social support, and emotional symptoms. The findings are expected to provide empirical evidence for developing regional social functioning rehabilitation programs, optimizing the allocation of community mental health resources, and improving patients’ quality of life and level of social integration.

## 2 Methods

### 2.1 Participants

In 2022, we performed a district-wide, census-based cross-sectional survey covering all registered patients with SMI in Minhang District, Shanghai.

#### 2.1.1 Inclusion criteria

1. Met the diagnostic criteria for the six categories of SMI based on the Diagnostic and Statistical Manual of Mental Disorders, 5th Edition (DSM-5);
2. Registered in the Shanghai Mental Health Information Management System;
3. Had resided in Minhang District for no less than six months;
4. Aged 18 years or older;
5. Able to complete the questionnaire independently or with assistance from family caregivers;
6. Provided written informed consent and agreed to participate in this survey.

#### 2.1.2 Exclusion criteria

1. Experiencing an acute psychiatric episode, or presenting with severe and refractory psychiatric symptoms (e.g., active hallucinations, delusions, marked apathy, disorganized behavior) or dangerous behaviors toward oneself or others;
2. Suffering from severe physical illnesses (e.g., severe cardiovascular and cerebrovascular diseases, malignant tumors);
3. Having severe cognitive impairment that prevented questionnaire completion;
4. Receiving long-term care in hospitals or nursing homes;
5. Planning to relocate out of Minhang District in the near future.

A total of 3835 questionnaires were distributed, and 3510 valid responses were finally included in the analysis. The enrolled participants were mostly patients with mild or stable psychiatric symptoms who could live in the community. The diagnostic distribution of the sample was as follows: schizophrenia (n = 2471, 70.40%), intellectual disability with mental disorders (n = 693, 19.74%), bipolar disorder (n = 250, 7.12%), epilepsy-related mental disorders (n = 81, 2.31%), paranoid psychosis (n = 9, < 1%), and schizoaffective disorder (n = 6, < 1%).

### 2.2 Research instruments

A general information questionnaire was developed by the research team to collect basic information, including age, gender, education level, marital status, occupation, disease duration, medication pattern, and medication adherence.

#### 2.2.1 The social disability screening schedule

Social functioning was assessed using the Social Disability Screening Schedule (SDSS) (2). This scale consists of 10 items, each scored on a 3-point scale (0 = no impairment, 1 = mild impairment, 2 = severe impairment), with higher total scores indicating more severe social dysfunction. The original scale has demonstrated good reliability and validity, with inter-rater agreement ranging from 85% to 99% and Kappa values between 0.6 and 1.0.Given that no standardized domain structure exists for the SDSS in large community-based samples of patients with SMI, the present study exploratively regrouped the 10 items into four functional domains based on item content and clinical relevance: personal life function (Item 8), family function (Items 2, 3, 6, and 7), social interaction (Items 4 and 5), and responsibility (Items 1 and 10).Univariate analyses (t-tests and ANOVA) of these domain scores were conducted for exploratory and descriptive purposes only. The primary outcome was the total SDSS score, which was used in all primary statistical analyses, including correlation and multiple linear regression.

Currently, limited studies have adopted this domain regrouping method. Therefore, we conducted psychometric analyses to evaluate its reliability and structural validity in our sample. Internal consistency was assessed using Cronbach’s α (Python). Among 3,510 valid samples, the overall SDSS showed good reliability (*α* = 0.87). The family function (*α* = 0.77) and social interaction (*α* = 0.77) domains reached acceptable levels. The personal life function domain consisted of a single item and therefore could not be evaluated with Cronbach’s α. The role functioning showed a Cronbach’s α of 0.33. Exploratory factor analysis (EFA) and confirmatory factor analysis (CFA) were conducted to examine the factor structure of the four domains. EFA supported a one-factor solution accounting for 71.88% of the total variance, with factor loadings ranging from 0.81 to 0.91. CFA confirmed good model fit: *χ²*/*df* = 1.82, RMSEA = 0.05, CFI = 0.96, TLI = 0.95, SRMR = 0.05. Composite reliability (CR = 0.90) and average variance extracted (AVE = 0.64) were satisfactory. **Table 1** summarizes the factor loadings from both EFA and CFA.

**Table 1:** Factor loadings of the regrouped SDSS domains.

Domain	EFA loading	CFA loading
Personal living	0.81	0.81
Family functioning	0.81	0.81
Social interaction	0.91	0.91
Responsibility	0.85	0.85

EFA, Exploratory Factor Analysis; CFA, Confirmatory Factor Analysis.

#### 2.2.2 The perceived social support scale

Perceived social support was measured using the Perceived Social Support Scale (PSSS) (3). The Chinese revised version developed by Jiang Qianjin was adopted in this study, which consists of 12 items distributed across three domains: family support, friend support, and other support, with four items for each domain. Specifically, family support includes items 3, 4, 8, and 11; friend support includes items 6, 7, 9, and 12; and other support includes items 1, 2, 5, and 10. Higher total scores indicate greater levels of perceived social support. This scale demonstrates satisfactory psychometric properties, with a Cronbach’s α coefficient of 0.88 and a test-retest reliability of 0.85. High inter-factor correlations (generally above 0.65) support its good structural validity.

#### 2.2.3 The generalized anxiety disorder 7-item

Anxiety symptoms were assessed using the Generalized Anxiety Disorder 7-item (GAD-7) scale (4), which evaluated the severity of anxiety symptoms. Scores were categorized as normal (0-4), mild (5-9), moderate (10-14), and severe (15-21). The Chinese version had good reliability and validity, with a Cronbach’s α of 0.91. A single-factor structure explained 64.7% of the variance. Using a cut-off value of 6, the sensitivity for screening generalized anxiety disorder was 0.86, specificity was 0.76, and the area under the ROC curve was 0.88.

#### 2.2.4 The patient health questionnaire 9-item

Depressive symptoms were assessed using the Patient Health Questionnaire 9-item (PHQ-9) scale (5), which evaluated the severity of depressive symptoms. Scores were categorized as normal (0-4), mild (5-9), moderate (10-14), and severe (15-27). The Chinese version had good reliability and validity, with a Cronbach’s α of 0.883 and good structural validity.

### 2.3 Data collection

The survey was conducted by community mental health staff who had received standardized training and served as investigators. Face-to-face interviews were used for data collection. Prior to the survey, all investigators were trained in the use of assessment scales and interview techniques to ensure data quality. A pilot study was also conducted to test the reliability and validity of the instruments. During the survey process, a double-checking procedure was implemented to ensure the accuracy of data entry. Patients with intact cognitive function completed face-to-face interviews, while assessments for those with cognitive impairment used information from their guardians.

### 2.4 Statistical Analysis

All data were analyzed using SPSS version 26.0. Continuous variables were expressed as mean ± standard deviation (Mean ± SD), and categorical variables were presented as frequencies and percentages [n (%)].For univariate analyses, independent-samples t-tests were used for comparisons between two groups, and one-way analysis of variance (ANOVA) was used for comparisons among multiple groups. To control for Type I error due to multiple comparisons, Bonferroni correction was applied, with the adjusted significance level calculated as α′ = *α*/*k* (where *α* = 0.05 and *k* represents the number of independent tests within the same domain).

Multiple linear regression analysis was performed to examine the independent effects of influencing factors on social functioning. The total SDSS score was set as the dependent variable. Age and gender were included as control variables, while total perceived social support score, anxiety score, depression score, disease duration, and medication adherence were included as independent variables. Multicollinearity was assessed using the variance inflation factor (VIF), with VIF < 5 indicating no multicollinearity and VIF ≥ 10 indicating severe multicollinearity. Pearson correlation analysis was conducted to examine the relationships among variables. A two-tailed *P* value < 0.05 was considered statistically significant. Python was used for multicollinearity testing and supplementary regression analyses. Subgroup analyses stratified by six diagnostic categories were performed to explore diagnostic heterogeneity and verify the robustness and consistency of the main associations across different diagnostic subgroups.

### 2.5 Ethics approval

This study was approved by the Ethics Committee of Shanghai Mental Health Center Pujiang Hospital (approval No. LW202526).All procedures performed were in accordance with the 1964 Helsinki Declaration and its later amendments. Written informed consent was obtained from all participants or their legal guardians.

## 3 Results

### 3.1 General clinical and functional characteristics

#### 3.1.1 General characteristics of the participants

The final sample consisted of 3510 patients with SMI, with slightly more females (51.30%) than males (48.70%). The mean age was 59.00 ± 14.70 years, and nearly half of participants were aged 60 years and older. Most respondents had a low educational level (primary school or below), while married and widowed status accounted for the majority in terms of marital status. The mean disease duration was 30.30 ± 16.90 years. In addition, 65.90% of participants maintained regular medication adherence. Detailed demographic and clinical characteristics are presented in **Table 2**.

**Table 2:** General characteristics of the study participants.

Characteristic	Category	Count (n)	Percentage (%)
Sex	Male	1709	48.70
	Female	1801	51.30
Age (years)	< 40	527	15.00
	40~59	1263	36.00
	60~79	1473	42.00
	≥ 80	247	7.00
Educational level	Primary school or below	2057	58.60
	Junior high school	983	28.00
	Senior high school or above	470	13.40
Marital status	Married	1351	38.50
	Widowed	1236	35.20
	Single	643	18.30
	Divorced	280	8.00
Medication status	Regular medication	2313	65.90
	Irregular/No medication	1197	34.10

#### 3.1.2 Overall status of social functioning

Among the 3510 participants, the overall prevalence of social dysfunction was 36.6% (95% CI: 35.00% – 38.20%). The total SDSS score averaged 2.45 ± 4.20 (median = 1.00). As shown in **Table 3**, most patients (63.40%) had intact social functioning, while the remaining participants presented with mild, moderate or severe functional impairment to varying degrees.

**Table 3:** Distribution of social functioning impairment levels.

Degree of social functioning Impairment	SDSS Score	Count (n)	Percentage (%)	Mean ± SD( ± s)
Normal	0	2225	63.40	0.00 ± 0.00
Mild impairment	1~3	896	25.50	1.87 ± 0.82
Moderate impairment	4~6	287	8.20	4.89 ± 0.76
Severe impairment	≥7	102	2.90	8.35 ± 1.24
Total	–	3510	100.00	2.45 ± 4.20

#### 3.1.3 Impairment across SDSS domains

Impairment levels varied across the four SDSS domains. The social interaction domain presented the highest mean score (1.28 ± 2.03) and impairment rate (41.80%), which was the most severely impaired domain. By contrast, the personal life function domain had the lowest mean score (0.22 ± 0.49) and impairment rate (8.70%). Detailed scores and impairment rates for each domain are summarized in **Table 4**.

**Table 4:** SDSS domain scores and impairment rates.

SDSS domain	Mean ± SD	Impaired cases (n)	Impairment rate (%)	Mild impairment (%)	Severe impairment (%)
Personal life	0.22 ± 0.49	129	8.70	6.20	2.50
Family function	0.51 ± 1.58	265	17.90	12.30	5.60
Social interaction	1.28 ± 2.03	620	41.80	28.50	13.30
Responsibility capacity	0.52 ± 1.07	271	18.30	13.10	5.20

Impairment rate refers to participants with a score ≥ 1; mild impairment is defined as a score = 1, and severe impairment as a score ≥ 2.

#### 3.1.4 Prevalence of emotional symptoms

All 3510 participants were screened for anxiety and depressive symptoms. Overall symptom prevalence was defined as the proportion of individuals with mild, moderate or severe symptoms.

Most participants had no anxiety symptoms (84.90%), and the overall prevalence of anxiety symptoms was 15.10%. Depressive symptoms were more common, with an overall prevalence of 21.00%. In total, 11.30% of participants had comorbid anxiety and depressive symptoms. Detailed stratified data by symptom severity are presented in **Table 5**.

**Table 5:** Distribution of anxiety and depressive symptoms.

Symptom type	Severity	No. (n)	Percentage (%)	Total incidence (%)
Anxiety symptoms	Normal	2979	84.90	15.10
	Mild	291	8.30	
	Moderate	113	3.20	
	Severe	127	3.60	
Depressive symptoms	Normal	2773	79.00	21.00
	Mild	405	11.50	
	Moderate	168	4.80	
	Severe	164	4.70	
Anxiety & Depressive symptoms	–	397	11.30	11.30

Overall symptom prevalence was calculated as (number of participants with mild, moderate or severe symptoms/total sample) × 100%.

### 3.2 Effects of Different factors on social functioning (after bonferroni correction)

We analyzed the associations between social functioning and multiple influencing factors, including sex, age, educational level, marital status, medication status, emotional symptoms, social support and disease duration. Bonferroni correction was used to control the inflation of Type I error caused by multiple comparisons. The corrected significance level was calculated as *α′* = *α*/*k*, where *α* = 0.05 and k indicates the number of independent tests in each analysis. A *P* value below the corrected α′ was considered statistically significant.

#### 3.2.1 Gender differences in social functioning

Independent-samples t-tests were performed to compare SDSS and PSSS scores between male and female participants. No significant intergroup differences were observed in the total SDSS score, total PSSS score, or scores for the personal life, family function and social interaction domains (all *P* > 0.05). A significant difference was only detected in the responsibility capacity domain: females had lower scores than males (*P* = 0.02). Comparisons of all indicators by sex are presented in **Table 6**.

**Table 6:** Comparison of social functioning indicators by sex (Mean ± SD).

Indicator	Female (n = 1801)	Male (n = 1709)	t	*p*
SDSS total score	2.38 ± 4.28	2.52 ± 4.10	0.96	0.34
Personal life	0.21 ± 0.49	0.22 ± 0.50	0.45	0.65
Family function	0.51 ± 1.78	0.51 ± 1.32	0.12	0.90
Social interaction	1.25 ± 2.05	1.31 ± 2.01	0.83	0.41
Responsibility capacity	0.48 ± 1.01	0.56 ± 1.13	2.30	0.02
PSSS total score	53.42 ± 13.01	53.53 ± 12.92	0.26	0.80

Independent-samples t-test was applied. n = sample size. Statistical significance was set at *P* < 0.05.

#### 3.2.2 Social functioning by educational level

Participants were stratified into two groups according to educational level: ≤ 9 years and 9–12 years of schooling. Independent-samples t-tests were used to compare social functioning and perceived social support across groups. No significant differences were observed in the total SDSS score, scores for each SDSS domain, or total PSSS score (all *P* > 0.05). Detailed results are shown in **Table 7**.

**Table 7:** Comparison of social functioning indicators by educational level (mean ± SD).

Indicator	≤9years (n = 2767)	9~12years (n = 743)	t	*p*
SDSS total score	2.41 ± 4.04	2.59 ± 4.74	-0.91	0.37
Personal life	0.22 ± 0.49	0.22 ± 0.51	-0.36	0.72
Family function	0.50 ± 1.50	0.56 ± 1.87	-0.81	0.42
Social interaction	1.26 ± 2.00	1.35 ± 2.11	-0.96	0.34
Responsibility capacity	0.51 ± 1.01	0.56 ± 1.27	-0.97	0.33
PSSS total score	53.43 ± 12.97	53.61 ± 12.92	-0.33	0.75

Independent-samples t-test was applied. n = sample size. Statistical significance was set at *P* < 0.05.

#### 3.2.3 Social functioning by marital status

Participants were stratified into four groups based on marital status: married, single, divorced and widowed. One-way ANOVA was applied to compare social functioning and perceived social support across groups. No significant intergroup differences were found in the total SDSS score, each SDSS domain score or total PSSS score (all *P* > 0.05). Detailed results are presented in **Table 8**.

**Table 8:** Comparison of social functioning indicators by marital status (mean ± SD).

Indicator	Married	Widowed	Single	Divorced	F	*p*
SDSS total score	2.43 ± 4.43	2.46 ± 4.01	2.54 ± 3.90	2.41 ± 3.75	0.07	0.98
Personal life	0.20 ± 0.47	0.22 ± 0.51	0.24 ± 0.50	0.27 ± 0.54	1.39	0.24
Family function	0.53 ± 1.81	0.50 ± 1.33	0.50 ± 1.43	0.38 ± 0.81	0.47	0.70
Social interaction	1.24 ± 2.03	1.30 ± 2.04	1.37 ± 1.95	1.33 ± 2.10	0.51	0.68
Responsibility capacity	0.52 ± 1.14	0.52 ± 0.98	0.56 ± 1.15	0.51 ± 0.85	0.14	0.94
PSSS total score	53.56 ± 12.98	53.32 ± 12.95	53.28 ± 13.24	54.18 ± 12.30	0.27	0.85

One-way analysis of variance (ANOVA) was used. Statistical significance was set at *P* < 0.05.

#### 3.2.4 Social functioning by medication status

Participants were stratified into two groups based on medication adherence: regular medication and irregular/no medication. Independent-samples t-tests were performed to compare social functioning and perceived social support. No significant differences were found in the total SDSS score, total PSSS score, or most SDSS domains (all *P* > 0.05). A significant difference was only observed in the social interaction domain, where participants on regular medication obtained higher scores than those with irregular or no medication (*P* = 0.03). Full results are presented in **Table 9**.

**Table 9:** Comparison of social functioning indicators by medication status (Mean ± SD).

Indicator	Irregular/No medication	Regular medication	t	*p*
SDSS total score	2.33 ± 4.07	2.51 ± 4.26	-1.20	0.23
Personal life	0.20 ± 0.48	0.23 ± 0.50	-1.60	0.11
Family function	0.53 ± 1.72	0.50 ± 1.51	0.46	0.65
Social interaction	1.18 ± 1.88	1.32 ± 2.10	-2.15	0.03
Responsibility capacity	0.52 ± 1.08	0.52 ± 1.07	-0.22	0.83

Independent-samples t-test was applied. Statistical significance was set at *P* < 0.05.

#### 3.2.5 Social functioning by age group

Participants were stratified into four age groups: < 40 years, 40–59 years, 60–79 years and ≥ 80 years. One-way ANOVA was used to compare social functioning and perceived social support across groups. Following Bonferroni correction (*α′* = 0.008), a significant group difference was detected only in the personal life domain (*F* = 7.94, *P* < 0.001). Participants aged ≥ 80 years had the highest score (0.31 ± 0.59), whereas those aged < 40 years had the lowest (0.13 ± 0.37). No significant differences were found for the remaining domains (all *P* > 0.008). Detailed data are shown in **Table 10**.

**Table 10:** Comparison of social functioning indicators by age group (mean ± SD).

Indicator	≤ 40 years	40–59 years	60–79 years	≥ 80 years	F	*p*
SDSS total score	2.04 ± 3.89	2.54 ± 4.31	2.48 ± 4.20	2.47 ± 4.07	1.47	0.22
Personal life	0.13 ± 0.37	0.24 ± 0.52	0.21 ± 0.48	0.31 ± 0.59	7.94	< 0.001
Family function	0.51 ± 1.67	0.59 ± 1.97	0.45 ± 1.29	0.54 ± 1.19	1.76	0.15
Social interaction	1.02 ± 1.82	1.31 ± 2.02	1.33 ± 2.07	1.19 ± 1.98	2.68	0.05
Responsibility capacity	0.47 ± 1.06	0.52 ± 1.03	0.53 ± 1.09	0.57 ± 1.22	0.49	0.69
PSSS total score	53.48 ± 12.70	53.39 ± 13.16	53.43 ± 12.91	54.27 ± 12.67	0.27	0.84

One-way analysis of variance (ANOVA) was employed. The significance level was set at *P* < 0.008 after Bonferroni correction.

#### 3.2.6 Effects of emotional symptoms on social functioning

**Figure 1:**
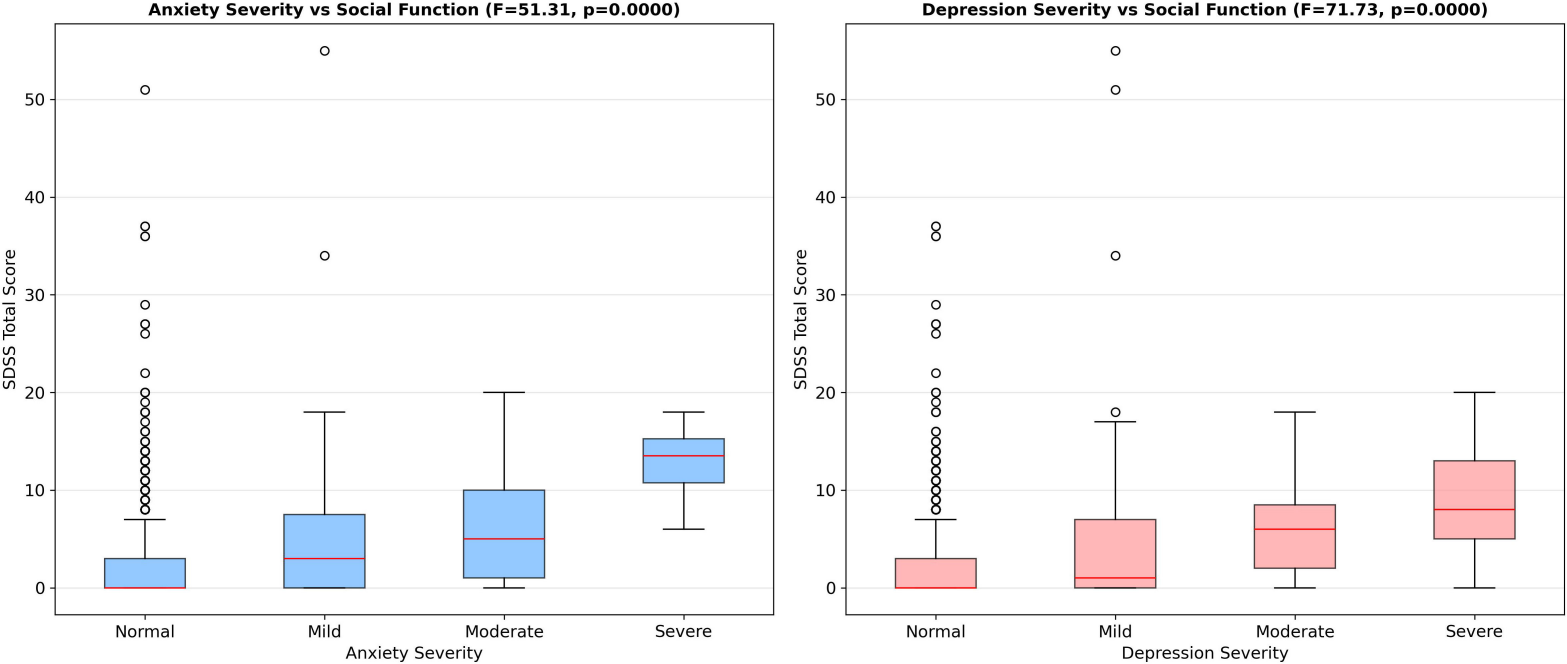
The impact of different levels of anxiety and depressive symptoms on social functioning.

We further compared total SDSS scores across participants with different severities of anxiety and depressive symptoms. According to GAD-7 and PHQ-9 results, symptoms were classified into three levels: normal, mild, and moderate or above. After Bonferroni correction (*α′* = 0.025, *k* = 2), participants with moderate or more severe anxiety symptoms presented higher total SDSS scores than those with normal anxiety (*F* = 58.36, *P* < 0.001). A similar trend was observed for depressive symptoms: participants with moderate or above depressive symptoms also had significantly higher SDSS scores relative to the normal group (*F* = 72.54, *P* < 0.001). Detailed results are shown in **Table 11**; **Figure 1**.

**Table 11:** Comparison of SDSS total scores by levels of emotional symptoms (mean ± SD).

Symptom type	Severity	SDSS total score	*t/F*	*P*
Anxiety Symptoms	Normal	2.32 ± 4.05	58.36	< 0.001
	Mild	4.33 ± 4.82		
	Moderate or above	6.31 ± 5.26		
Depressive Symptoms	Normal	2.18 ± 3.98	72.54	< 0.001
	Mild	4.56 ± 4.91		
	Moderate or above	6.72 ± 5.38		

One-way ANOVA was performed. The corrected significance level was set at *P* < 0.025 after Bonferroni correction.

#### 3.2.7 Relationship between social support and social functioning

**Figure 2:**
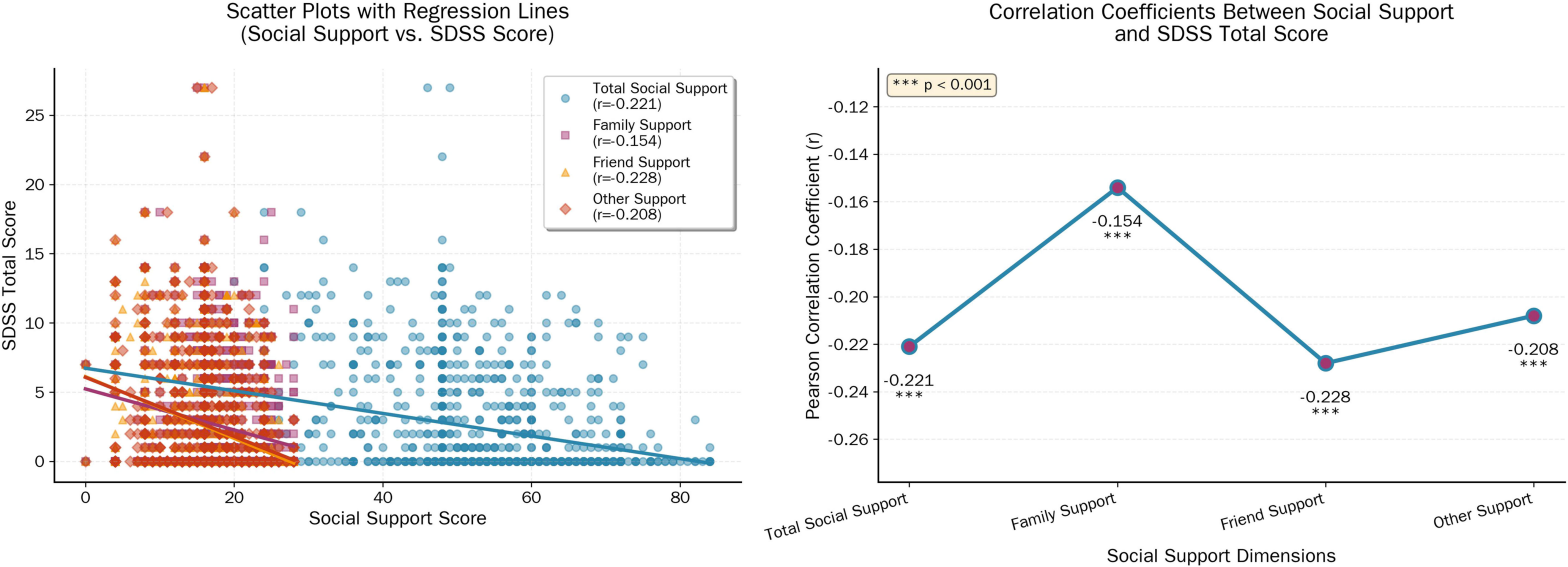
The impact of friend support, family support, other support and total PSSS score on social functioning.*** indicates statistically significant difference at *p* < 0.001.

After Bonferroni correction (*α′* = 0.0125, *k* = 4), Pearson correlation analyses revealed that the total PSSS score and its three subdomains (family support, friend support and other support) were significantly and negatively correlated with the total SDSS score (all *P* < 0.001). Correlation coefficients ranged from *r* = −0.15 to −0.23, indicating weak correlations. Higher levels of social support corresponded to milder social functioning impairment. Despite the weak correlation magnitudes, the findings were robust given the large sample size. Detailed correlation results are presented in **Table 12**; **Figure 2**.

**Table 12:** Scores of social support domains and their correlation with social functioning.

Variable	Total PSSS score	Family support	Friend support	Other support	Total SDSS score
Total PSSS Score	1.00	_	_	_	_
Family Support	0.82	1.00	_	_	_
Friend Support	0.92	0.58	1.00	_	_
Other Support	0.94	0.63	0.88	1.00	_
Total SDSS Score	-0.22***	-0.15***	-0.23***	-0.21***	1.00

Pearson correlation analysis was applied. ***P < 0.001 after Bonferroni correction.

#### 3.2.8 Relationship between disease duration and social functioning

Participants were divided into three groups according to disease duration: < 10 years, 10–30 years and > 30 years. One-way ANOVA was used to compare total SDSS scores across groups. The mean total SDSS scores were 1.92 ± 3.58 for the < 10 years group, 2.49 ± 4.15 for the 10–30 years group, and 3.15 ± 4.67 for the > 30 years group. A significant intergroup difference was identified (*F* = 6.28, *P* < 0.001). Detailed results are presented in **Table 13**.

**Table 13:** Comparison of SDSS total scores by disease duration(mean ± SD).

Disease duration (years)	Cases (n)	SDSS total score	*F*	*P*
< 10	782	1.92 ± 3.58	6.28	< 0.001
10 - 30	1645	2.49 ± 4.15		
> 30	1083	3.15 ± 4.67		

One-way analysis of variance (ANOVA) was applied.

### 3.3 Multiple linear regression analysis of factors influencing social functioning

#### 3.3.1 Overall model fit

We first tested multicollinearity among all independent variables before performing multiple linear regression. The variance inflation factor (VIF) was 1.28 for age, 1.01 for sex, 1.06 for total PSSS score, 2.12 for total GAD score, 2.15 for total PHQ score, 1.05 for medication adherence and 1.34 for disease duration. All VIF values were within the acceptable range, suggesting no severe multicollinearity.

The multiple linear regression model was established using the total SDSS score as the dependent variable. Age and sex were entered as covariates, whereas total PSSS score, total GAD score, total PHQ score, disease duration and medication adherence were defined as independent variables. The overall model was significant (*F* = 68.55, *P* < 0.001), with an adjusted *R²* of 0.119.

#### 3.3.2 Independent effects of influencing factors

**Figure 3:**
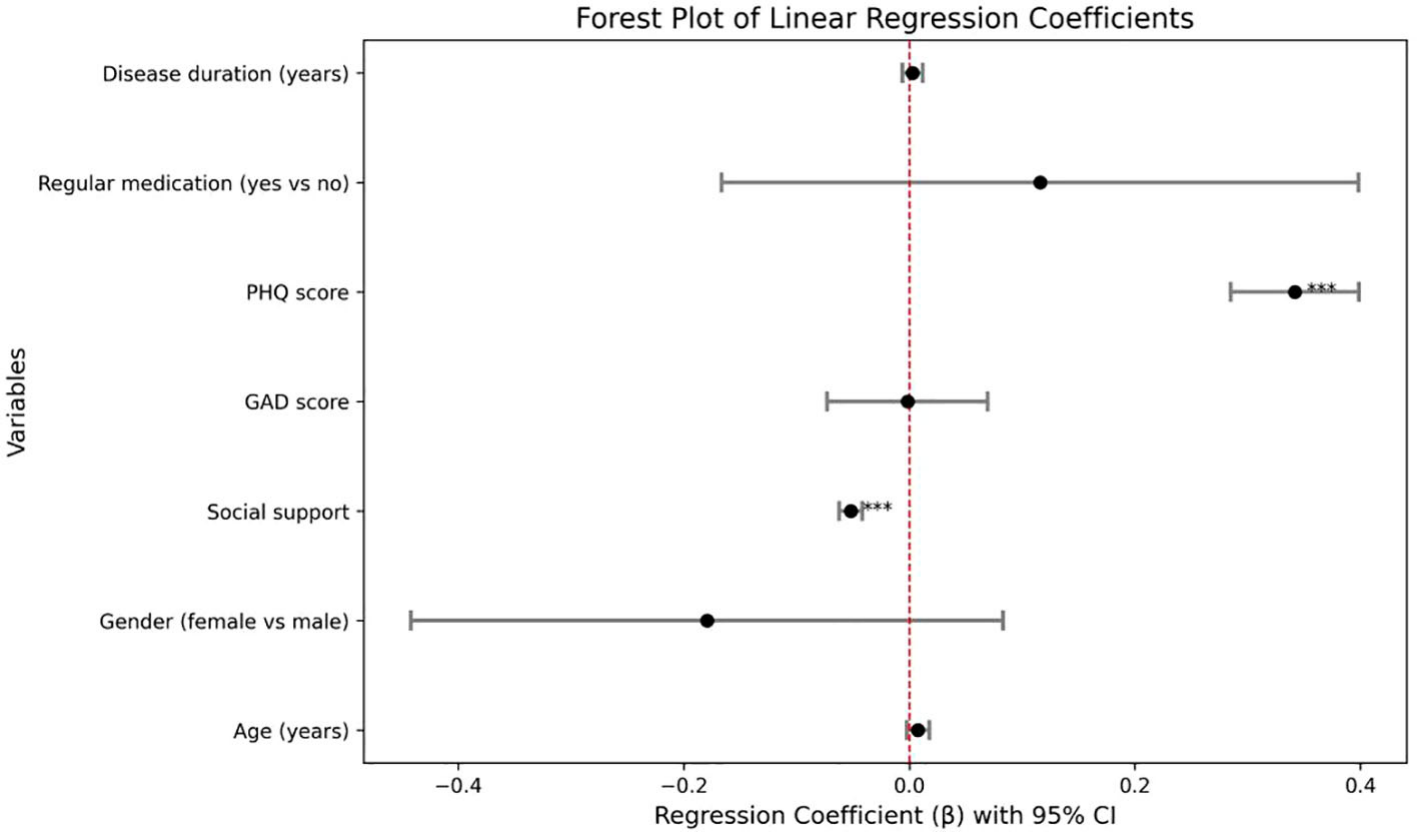
Results of multiple linear regression analysis on influencing factors of social functioning.

After adjusting for age and sex, only two variables exerted significant independent effects on social functioning (both *P* < 0.001). The total PHQ score was positively associated with the total SDSS score (*β* = 0.27, *P* < 0.001), indicating that more severe depressive symptoms corresponded to worse social functioning. Conversely, the total PSSS score was negatively correlated with the total SDSS score (*β* = -0.16, *P* < 0.001), suggesting that higher perceived social support was linked to better social functioning. No significant independent effects were observed for the remaining variables (*P* = 0.14,*P* = 0.18,*P* = 0.97,*P* = 0.42,*P* = 0.52) (**Figure 3**, **Table 14**).

**Table 14:** Multiple linear regression analysis of factors influencing social functioning.

Variable name	Unstandardized coefficient (*B*)	Standardized coefficient (*β*)	*t*	*P*	95% CI
Intercept	3.88	0.00	9.16	< 0.001	[3.05, 4.72]
Age (years)	0.01	0.03	1.48	0.14	[-0.00, 0.02]
Sex	-0.18	-0.04	-1.34	0.18	[-0.04, 0.08]
PSSS	-0.05	-0.16	-9.84	< 0.001	[-0.06, -0.04]
PHQ	0.34	0.27	11.74	< 0.001	[0.29, 0.40]
GAD	-0.00	-0.00	-0.05	0.97	[-0.07, 0.07]
Medication regularity	0.12	0.03	0.81	0.42	[-0.17,0.40]
Disease duration	0.00	0.01	0.64	0.52	[-0.01,0.01]

P < 0.001 was considered statistically significant. Standardized coefficient (β) was used to compare the relative magnitude of associations. Variable coding: Sex, 0 = male; Medication regularity, 0 = no medication. Disease duration was measured in years.

### 3.4 Subgroup analysis by diagnostic category

We conducted subgroup analyses across six diagnostic groups to examine the impact of diagnostic heterogeneity. Significant intergroup differences were observed for age (*F* = 23.43, *P* < 0.001) and illness duration (*F* = 92.72, *P* < 0.001). However, no significant differences were found among diagnostic subgroups in the total scores of SDSS, PSSS, GAD-7, or PHQ-9 (*P* = 0.98, *P* = 0.43, *P* = 0.07, *P* = 0.35, respectively). Exact *P*-values and sample sizes for each subgroup are reported in **Table 15**.

**Table 15:** Subgroup characteristics and key outcomes by diagnostic category (mean ± SD).

Diagnostic category	N	Age(years)	Duration(years)	SDSS total	PSSS total	GAD-7	PHQ-9
Schizophrenia	2471	60.60 ± 13.80	28.90 ± 16.10	2.46 ± 4.16	53.71 ± 12.92	1.82 ± 2.81	2.15 ± 3.41
Intellectual disability accompanied by mental disorders	693	55.10 ± 14.90	39.70 ± 18.20	2.47 ± 4.18	53.32 ± 13.22	1.95 ± 2.93	2.31 ± 3.57
Bipolar disorder	250	53.80 ± 15.60	16.60 ± 12.30	2.36 ± 3.72	52.21 ± 12.80	2.28 ± 3.14	2.56 ± 3.82
Mental disorders due to epilepsy	81	62.60 ± 12.70	36.10 ± 17.50	2.43 ± 6.56	52.81 ± 13.00	2.17 ± 3.05	2.43 ± 3.69
Paranoid psychosis	9	62.00 ± 11.50	28.20 ± 15.80	1.44 ± 3.36	48.40 ± 11.70	1.67 ± 2.64	1.89 ± 3.15
Schizoaffective disorder	6	56.50 ± 13.10	24.30 ± 14.60	2.00 ± 2.76	52.51 ± 12.50	2.00 ± 2.87	2.17 ± 3.38
*F*	—	23.43	92.72	0.15	0.98	2.00	1.12
*P*	—	< 0.001	< 0.001	0.98	0.43	0.07	0.35

One-way ANOVA was used for intergroup comparisons. Exact P values are presented. SDSS = Social Disability Screening Schedule; PSSS = Perceived Social Support Scale; GAD-7 = 7-item Generalized Anxiety Disorder scale; PHQ-9 = 9-item Patient Health Questionnaire.

Despite differences in age and illness duration, the overall severity of social dysfunction, social support, anxiety, and depression was comparable across diagnostic groups. Furthermore, the correlation patterns between depressive symptoms, social support, and social functioning were consistent across all subgroups: more severe depressive symptoms were associated with poorer social functioning, whereas higher social support was linked to better social functioning. These consistent findings suggest that the primary results are robust and not influenced by diagnostic heterogeneity.

## 4 Discussion

### 4.1 Current Status of social functioning among SMI patients in Minhang District

The results of this study show that the prevalence of social dysfunction among SMI patients in Minhang District was 36.6%, which was markedly lower than a rate of 73.9% reported for individuals with schizophrenia in a previous Beijing-based investigation (6). This discrepancy is primarily explained by differences in study populations. Earlier research generally targeted a single diagnostic group, namely patients with schizophrenia, while the current study enrolled participants across six SMI subtypes diagnosed in accordance with the DSM-5 criteria. The severity of social dysfunction varied considerably across diagnostic categories.

In addition, Minhang District has established a standardized community mental health management system based on the “Shanghai model of a three-tier mental health prevention and treatment network” proposed by Professor Zhang Mingyuan (7). This framework facilitates the delivery of clinical treatment, routine care and basic rehabilitation services on an ongoing basis. These findings indicate that a well-established, standardized community management model can deliver comprehensive supportive interventions. Such services help preserve patients’ overall social functioning and may account for the relatively low prevalence of social dysfunction in this area.

Stratified analyses across specific social functioning domains revealed an obvious imbalance: social interaction was the most impaired domain, while personal life showed the mildest impairment. On the one hand, the more severe impairment in social interaction may be related to the mediating role of stigma between illness and social isolation (8). Previous studies have shown that stigma is significantly associated with social alienation among SMI patients and serves as an independent influencing factor (9). Moreover, the mediating pathways of stigma are complex, involving chain mediation (10) and partial mediation effects (11). Empirical evidence from international studies and theoretical models (12–14) further supports the mediating role of stigma, while the “rejection–withdrawal” vicious cycle identified at the behavioral level (15) provides additional explanation for the formation of social isolation.

On the other hand, the relatively mild impairment in personal life function may benefit from the continuity of family caregiving and the availability of community-based daily care services, which provide strong support for patients’ basic self-care abilities. This phenomenon also reflects the priority placed on daily living support within national clinical and management guidelines for SMI. Collectively, these results suggest that although most patients can maintain basic activities of daily living, the inherent features of severe mental illness often lead to impaired social skills. As a result, patients struggle to establish and sustain normal social connections and social roles. The above observations are in line with conclusions from prior studies (11, 16).

### 4.2 Relationship between different factors and social functioning

In the group comparison analyses, age was significantly associated with social functioning, particularly within the personal life domain. Older patients exhibited greater impairment in personal life function, which may be associated with age-related physical decline, gradual deterioration in cognitive function, and contraction of social support networks in some individuals (17, 18). In contrast, younger patients showed relatively better personal life function, suggesting advantages in physiological function and self-care ability, as well as greater potential for recovery of social functioning (19, 20). This finding is consistent with previous studies examining the relationship between age and social functioning in SMI patients (21, 22).

In this cohort, depressive symptoms were more prevalent and severe than anxiety symptoms among Minhang District SMI patients, indicating that depression is more prominent in this population. Comorbid anxiety and depressive symptoms were also prevalent. This finding is consistent with the results reported by Duan et al. in eastern China (23).To further control for confounding factors and identify independent influencing factors of social functioning, multiple linear regression analysis was conducted. The multiple linear regression accounted for 11.9% of the variance in social functioning. While this explanatory power is generally acceptable in psychosocial research, it implies that additional unmeasured variables contribute to social functioning. Several relevant factors not incorporated into our analysis, including psychiatric symptom severity, stigma levels and the use of community rehabilitation services, may also play a role (8, 24). The cross-sectional design of this study precludes any causal inference. Therefore, longitudinal studies are needed to examine the dynamic changes in social functioning, explore potential mediating and moderating mechanisms with a broader set of variables, thereby incorporating these variables to improve the predictive model.

The regression results showed that, after controlling for age and gender, more severe depressive symptoms were associated with greater impairment in social functioning, whereas higher levels of social support were associated with less impairment. This finding further confirms the associations observed in univariate analyses and a significant association was found between depressive symptoms, social support and social functioning after adjusting for other variables. It is noteworthy that anxiety symptoms, which showed significant associations in univariate analyses, did not demonstrate independent significance in the multivariate model. This may be related to the diagnostic composition of the sample. In this study, patients with schizophrenia accounted for a large proportion of the sample, and this disorder is more likely to be accompanied by depressive symptoms in clinical practice, whereas anxiety symptoms are relatively less common and often secondary manifestations. This clinical characteristic may, to some extent, attenuate the association between anxiety symptoms and social functioning.

Social support was significantly negatively correlated with social functioning impairment: higher social support was associated with milder functional deficits. This finding supports the protective effect of social support among individuals with mental disorders reported by O’Connor (25). The results of the present study indicate that higher levels of family support, friend support, and other support are associated with better social functioning, with all three showing a consistent direction of effect. These findings suggest that different domains of social support are stably associated with better social functioning, correlating with less severe functional impairment. However, given the cross-sectional design of this study, the relationship between social support and social functioning should be understood as an association rather than a causal relationship. Specifically, the relationship between social support and social functioning may be bidirectional. While we interpret social support as a protective factor, it is equally plausible that patients with better social functioning are more likely to obtain stronger social support. Therefore, the possibility of reverse causality cannot be excluded, and our cross-sectional design cannot determine the temporal directionality of this relationship. Future longitudinal studies are warranted to clarify the causal pathways between social support and social functioning.

SMI patients often have relatively narrow social networks, which may be related to impaired social interaction skills and social stigma. The lack of friend support may further exacerbate the deterioration of social interaction skills, creating a vicious cycle. Rodolico A (26) proposed that stable and balanced family support is closely associated with favorable social rehabilitation among individuals with mental disorders—a finding consistent with the present study, which also finds family support closely associated with better social functioning among patients with SMI, and aligns with the central role of the family in patient care within traditional Chinese culture. Among different sources of social support, friend support was more strongly associated with better outcomes, suggesting that peer interaction and support may be particularly valuable in maintaining patients’ social functioning and reducing social functional deficits. In contrast, although family support is also suggested to have a protective role, its correlation is relatively weaker, possibly because friend support is more strongly associated with social participation and interpersonal interaction. It is also plausible that patients with better social functioning are more capable of establishing and maintaining peer relationships, which may further contribute to their social functioning, indicating a bidirectional relationship. Future longitudinal studies are warranted to clarify the causal pathways between social support and social functioning. These findings suggest that interventions aimed at improving patients’ social functioning should not only strengthen family support but also prioritize the development of robust peer and friend support systems to promote social recovery more effectively.

Regarding the impact of medication adherence on social functioning, no statistically significant difference was found between the regular medication group and the irregular/no medication group in the total SDSS score. Only a statistically significant difference was observed in social interaction ability, and this difference was considered small in the present study. Although this finding appears inconsistent with previous studies emphasizing the role of pharmacological treatment in functional recovery (27), it may be explained by the characteristics of the study population. First, the irregular/no medication group included both patients with poor adherence and those who discontinued medication due to stable clinical conditions. For example, some patients with bipolar disorder may not require continuous medication during remission, and some patients with schizophrenia may enter a stable recovery phase after standardized treatment and no longer require long-term medication. These patients may have similar levels of social functioning as those receiving regular medication, which could reduce observable group differences. Secondly, the study population was composed of community - registered patients. Generally, these patients had milder disease severity and were more likely to be in the stable or recovery stages, which is fundamentally different from hospitalized patients with more severe conditions. In such populations, the association between medication and social functioning is weaker, while the association between social functioning and rehabilitation interventions as well as social support is stronger. Third, Minhang District has established a standardized community mental health management system through expert consultation and multi-party collaboration. Through measures such as medication supervision, condition monitoring, management of adverse effects, and family caregiving guidance, this system improves service accessibility, reduces the impact of medication side effects, and enhances patients’ beliefs in treatment, thereby providing comprehensive support and helping maintain overall social functioning.

As for the dimensional division of the SDSS scale, despite the relatively low Cronbach’s α coefficient of the responsibility dimension in our sample, supplementary EFA and CFA analyses confirmed its favorable factor loading and good structural validity. For this reason, we fully retained the responsibility dimension without deleting any relevant items throughout statistical analysis. Keeping this dimension does not produce obvious adverse effects on the completeness of the SDSS scale and the comparability of assessment results across different research populations.

### 4.3 Limitations

This study has several limitations. First, the domain structure of the SDSS was empirically and exploratively derived and not based on a pre-existing theoretical or validated framework. Accordingly, domain-level results are presented for descriptive purposes only, and all primary conclusions were drawn based on the total SDSS score to ensure the robustness and reliability of the findings. Second, the study included six distinct diagnostic categories of severe mental illness, which may introduce clinical and prognostic heterogeneity. Although our subgroup analyses confirmed that the direction of associations was consistent across all diagnostic groups, pooling different disorders together may still obscure disease-specific characteristics and introduce potential bias. Such heterogeneity may affect the interpretation of findings and the internal validity of the main conclusions, which should be noted when applying the results. Third, due to its cross-sectional design, this study can only identify associations between variables and cannot establish causal relationships or determine temporal directionality. This is particularly relevant to the relationship between social support and social functioning, which may be bidirectional (as discussed above), and reverse causality cannot be ruled out. Fourth, all measures relied on self-reports from patients or reports from caregivers, which may introduce common method bias. This reliance on a single source of information may have inflated the observed associations between variables due to shared method variance. Additionally, self-reports may be influenced by patients’ insight into their own condition, memory recall, or social desirability, potentially affecting the accuracy of the reported data. Fifth, although our inclusion criteria explicitly excluded patients with acute episodes or severe refractory psychiatric symptoms, and the sample consisted primarily of clinically stable community-dwelling individuals, we did not formally assess symptom severity using standardized instruments (e.g., PANSS or BPRS). Consequently, we cannot fully exclude the possibility that residual symptom variability may have influenced the results. Sixth, this study did not assess several potentially important factors, including participation in vocational rehabilitation, use of community rehabilitation services, and levels of stigma. Consequently, the analysis of influencing factors remains incomplete.

### 4.4 Future directions

Future studies should incorporate direct assessments of psychiatric symptoms, employ objective indicators (e.g., occupational performance, records of social activities) or informant reports to enable multidimensional evaluation, and expand the range of variables to further elucidate the mechanisms underlying social dysfunction. Longitudinal designs are needed to examine the directionality of the observed associations and to evaluate the long-term effectiveness of the proposed interventions.

## 5 Conclusion

In conclusion, in this cross-sectional study of SMI patients in Minhang District, social dysfunction was relatively mild overall, with social interaction being the most impaired domain. Depressive symptom severity was positively associated with social dysfunction, while social support showed a negative association. Although significant intergroup differences existed in age and illness duration, the main multivariable model had already adjusted for age and gender, and the consistent direction of associations across all diagnostic subgroups further supports the robustness of our findings. Notably, the relationship between social support and social functioning may be bidirectional, although causal directionality cannot be inferred due to the cross-sectional design. These findings suggest that regular depressive symptom screening and community-based social cognitive skills training (SCST) (28) may serve as potential approaches relevant to better social functioning in this population. Future longitudinal studies are warranted to examine the directionality of these associations and evaluate the long-term effectiveness of the proposed interventions.

## Data Availability

The raw data supporting the conclusions of this article will be made available by the authors, without undue reservation.
